# A biodegradable microneedle sheet for intracorporeal topical hemostasis

**DOI:** 10.1038/s41598-020-75894-w

**Published:** 2020-11-02

**Authors:** Mao Yokoyama, Namie Chihara, Atsushi Tanaka, Yosuke Katayama, Akira Taruya, Yuko Ishida, Mitsuru Yuzaki, Kentaro Honda, Yoshiharu Nishimura, Toshikazu Kondo, Takashi Akasaka, Nobuhiro Kato

**Affiliations:** 1 Department of Cardiovascular Medicine, Wakayama Medical University, 811-1 Kimiidera, Wakayama, 641-8510 Japan; 2 Faculty of Biology-Oriented Science and Technology, Kindai University, 930 Nishimitani Kinokawa, Wakayama, 649-6493 Japan; 3 Department of Forensic Medicine, Wakayama Medical University, Wakayama, Japan; 4 Department of Thoracic and Cardiovascular Surgery, Wakayama Medical University, Wakayama, Japan

**Keywords:** Preclinical research, Biomaterials, Hepatology, Trauma, Biomedical engineering

## Abstract

Management of bleeding is critical for improving patient outcomes. While various hemostatic products are used in daily practice, technical improvement is still needed. To addresses this problem, we newly developed a microneedle hemostatic sheet based on microneedle technology. We demonstrated the unique features of this microneedle hemostatic sheet, including reduced hemostatic time, biodegradable polymer composition that allows intracorporeal use without increasing infectious risk incorporation of microneedles to fix the sheet to the wound even on the left ventricular wall of a swine while beating, and a mesh structure with flexibility comparable to that of bonding surgical tape and sufficient rigidity to penetrate human aorta tissue and swine left ventricular wall. One potential application of the microneedle hemostatic sheet is intracorporeal topical hemostasis for parenchymatous organs, large vessels, and heart wall during trauma or surgery, in addition to new, widespread applications.

## Introduction

Management of bleeding during surgery or trauma is essential for improving patient outcomes. Allogeneic blood product transfusion is primarily chosen for the treatment of major bleeding. However, increased transfusion of blood products is associated with significantly increased mortality and morbidity^1^. Various hemostatic products have been developed to minimize the transfusion of blood products^2,3^ by helping to improve hemostasis with a consequent decrease in postoperative complications and hospital costs^3^. However, technical improvement is still needed in the areas of efficacy, handling, risks of viral infection and other transferable diseases, and allergic reactions resulting from human hemostatic components^4^.

To addresses these problems, we have newly developed a "microneedle hemostatic sheet (MHS)" based on microneedle technology and which has several unique features. It is made from biodegradable polymer for intracorporeal use, features microneedles that fix the sheet with friction force, and is configured as a mesh structure with flexibility for intraoperative usage. This study developed the manufacturing process of MHS and investigated the biosafety and hemostatic ability of this MHS.

## Results

### Manufacturing process

**Figure 1:**
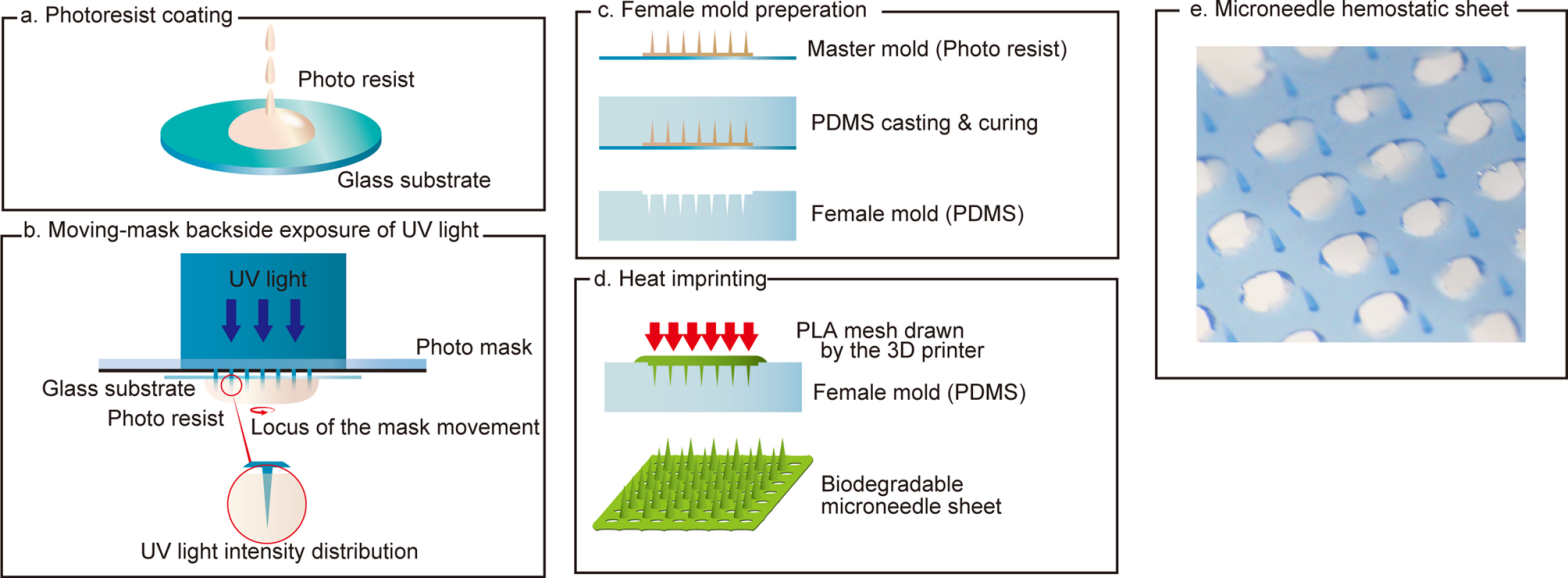
Microneedle hemostatic sheet manufacturing. (**a**) The photoresist coating process. (**b**) Moving-mask backside exposure of UV light. (**c**) Female mold preparation. (**d**) Heat imprinting process. (**e**) The microneedle hemostatic sheet.

The manufacturing process for the MHS is summarized in Fig. 1. On a glass substrate, a negative tone thick photoresist (SU-8 3050, KAYAKU Advanced Materials, Inc. Westborough, MA, USA) was dispensed thicker than 600 µm (Fig. 1a). After soft baking, the substrate was placed upside-down on the horizontal 2-axial piezoelectric movable stage of a specially made moving mask exposure apparatus. Through a photomask with a circular opening 90 µm in diameter and patterns measuring 17 mm × 17 mm with 1 mm spacing, UV light (λ = 365 nm) was illuminated on the substrate simultaneously with a 90-µm-diameter circular movement in plane at 0.5 Hz for 15 s. Consequently, the spatial UV-exposure dose was formed as a corn-shaped microneedle (Fig. 1b). Following post-exposure bake, development, rinse, and drying with nitrogen blow, a master mold of the microneedle was obtained^5,6^. The shape of the master mold was transferred by poly-dimethyl siloxane (PDMS) (Sylgard 184, Dow Corning Corp, Midland, MI, USA) to be a female mold (Fig. 1c). A three-dimensional (3D) printer (Trinus, Kodama, Inc. San Francisco, CA USA) was used to draw a poly-lactic acid (PLA) micro square mesh with a 200 µm-thick line of 1 mm pitch. The PLA micro mesh was aligned to the microneedle female mold under a stereomicroscope and covered with another PDMS slab. The mold set was set on a heat press machine (N4018-5 K-200, NPa system, Co., Ltd. Saitama, Japan) and pressed at 130 °C with 1 Pa pressure for 5 secs (Fig. 1d). After cooling, the MHS was obtained (Fig. 1e). The size of MHS was 20 mm × 20 mm. The height of microneedles was 500 µm.

### Flexibility measurement

**Figure 2:**
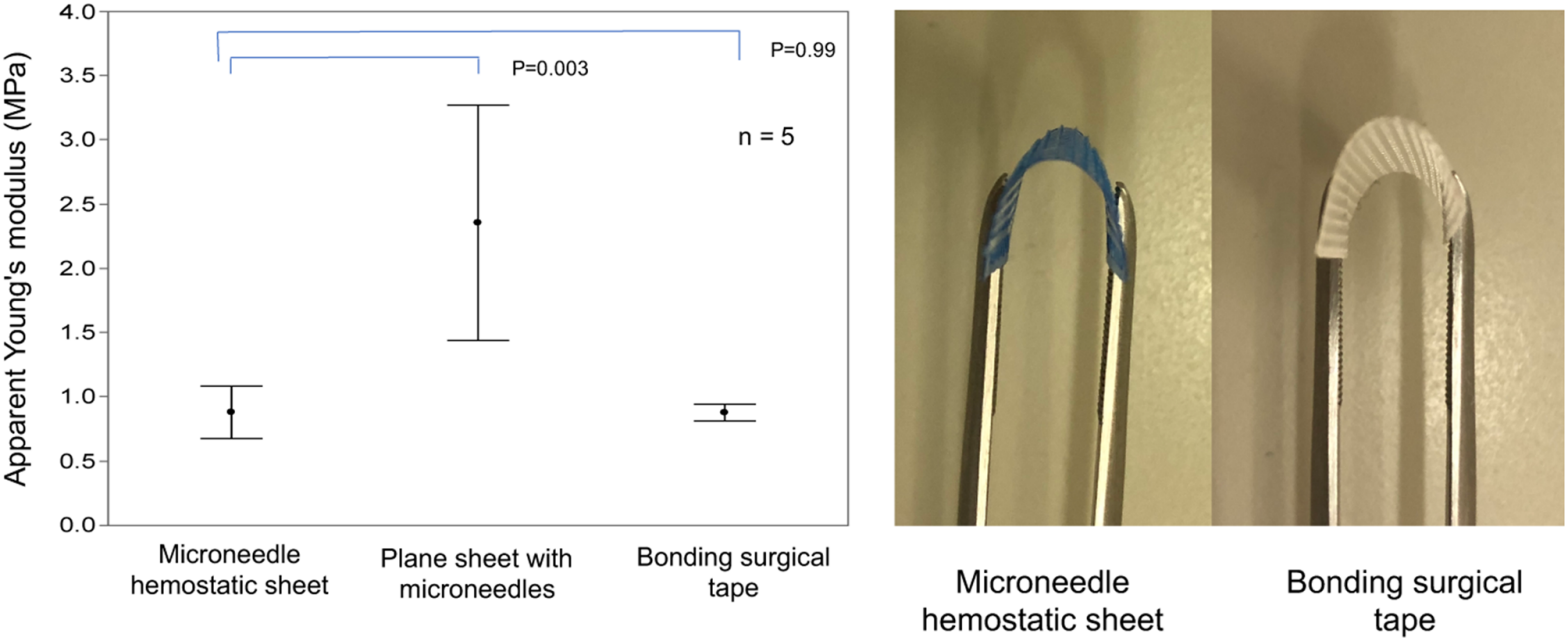
Results of flexibility measurements.

We applied the apparent Young's modulus for the flexibility of MHS. The results of the apparent Young's modulus measurements are presented in the left panel of Fig. 2. The MHS showed good flexibility compared to the plane sheet with microneedles; both were made of PLA and had the same thickness of 200 µm (MHS 0.881 ± 0.20 MPa vs. plane sheet with microneedles 2.356 ± 0.92 MPa, *p* = 0.003). The apparent Young's modulus was comparable between the MHS and the bonding surgical tape (0.881 ± 0.20 MPa vs. 0.877 ± 0.07 MPa, *p* = 0.999). The MHS was flexible enough to be bent with forceps and was comparable to that demonstrated by the bonding surgical tape (right panel of Fig. 2).

The left panel shows the results of flexibility test. The microneedle hemostatic sheet (MHS) shows good flexibility compared with the plane sheet with microneedles (0.881 ± 0.20 MPa vs. 2.356 ± 0.92 MPa, *p* = 0.003). The apparent Young's modulus is comparable between the MHS and the bonding surgical tape (0.881 ± 0.20 MPa vs 0.877 ± 0.07 MPa, *p* = 0.999). The MHS is flexible enough to be bent with forceps and is comparable to that demonstrated by the bonding surgical tape (right panel of Fig. 2).

### Hemostatic time in the mouse liver model

**Figure 3:**
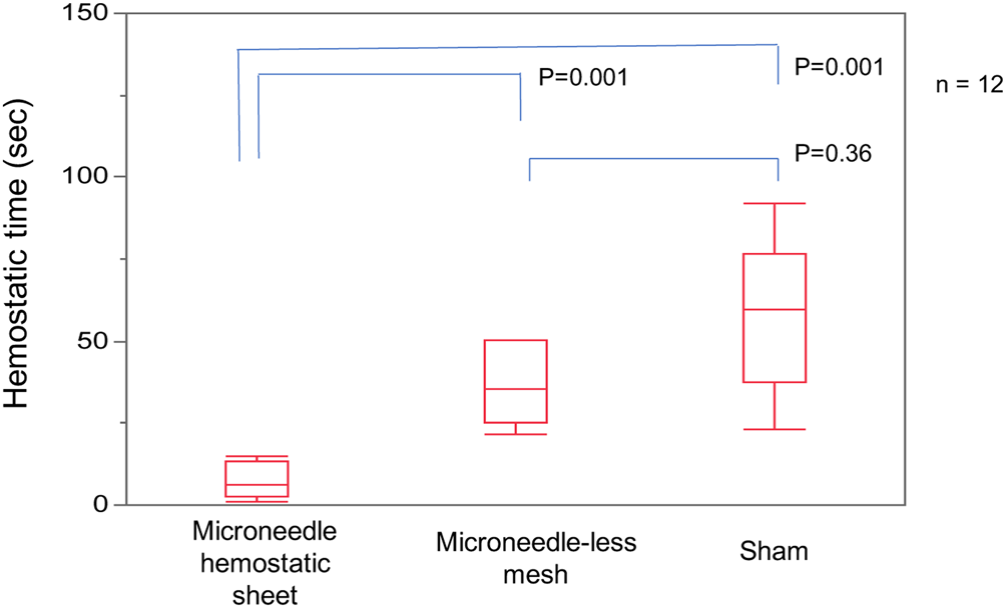
Hemostatic time in the mouse liver model.

The hemostatic times of each group in the mouse liver model are shown in Fig. 3. The time in the MHS group was significantly lower than that in the microneedle-less mesh and sham gssroups (MHS 6.2 [2.8–13.3] sec vs. microneedle-less mesh 35.4 [25.3–50.2] sec vs. sham 59.5 [37.4–6.6] sec], *p* = 0.00001). The Steel–Dwass test showed that the bleeding time of the microneedle sheet group was shorter than that of the other groups (MHS vs. microneedle-less mesh, *p* = 0.001; MHS vs. sham, *p* = 0.001), while no difference was observed between the microneedle-less mesh and sham groups (*p* = 0.36).

The hemostatic time is significantly reduced in the microneedle hemostatic sheet (MHS) group compared to those in the microneedle-less mesh and sham groups (MHS 6.2 [2.8–13.3] sec vs. microneedle-less mesh 35.4 [25.3–50.2] sec vs. sham 59.5 [37.4–76.6] sec], *p* = 0.00001). The bleeding time of the MHS group is shorter than those of other groups (MHS vs. microneedle-less mesh, *p* = 0.001, microneedle vs. sham 59.5, *p* = 0.001) while no difference is observed between the microneedle-less mesh and sham groups (*p* = 0.36).

### Pathological assessment of the microneedle hemostatic sheet

**Figure 4:**
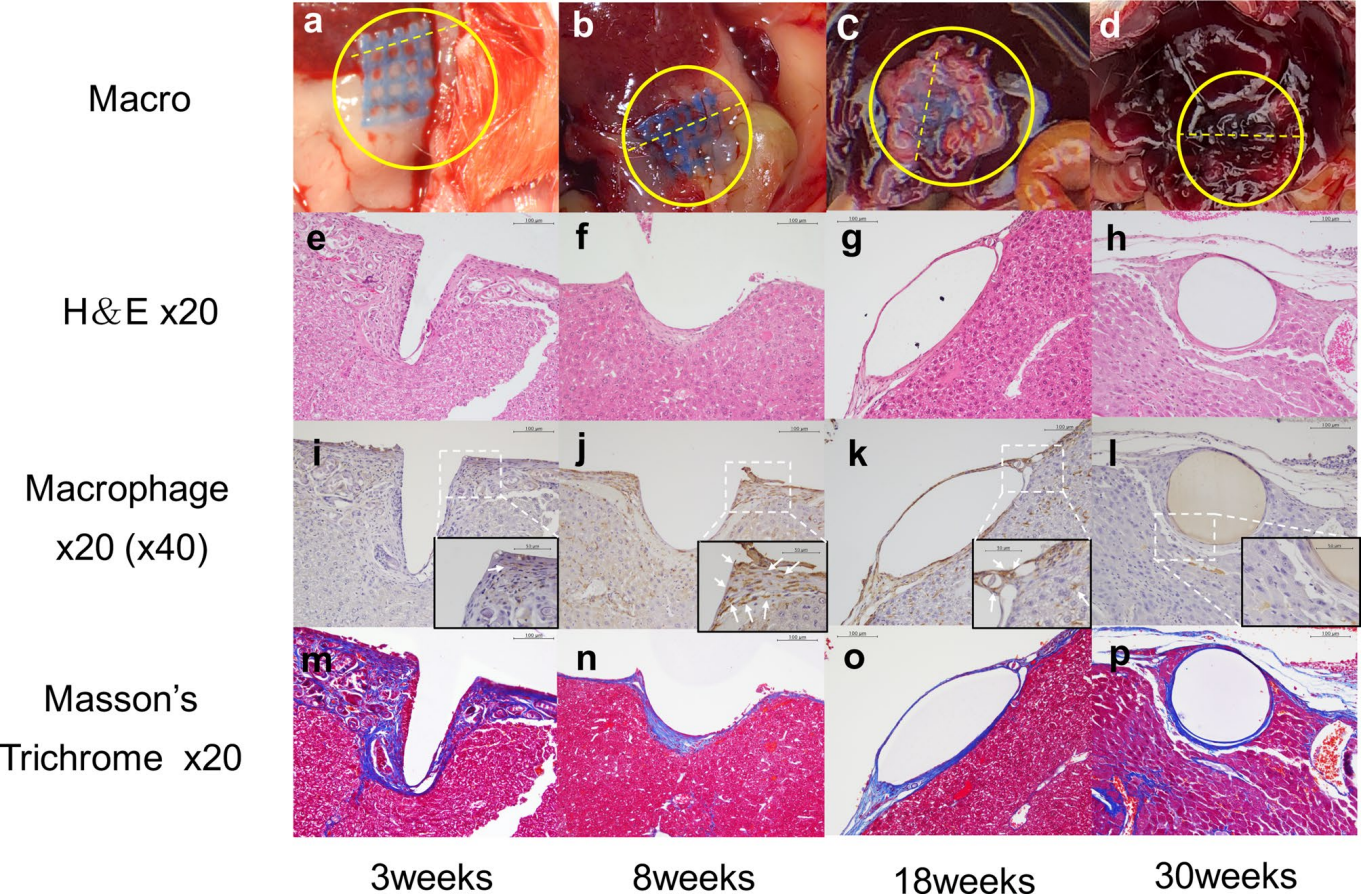
Pathological assessment of the microneedle hemostatic sheet after implantation. Macro-view at each point (**a**–**d**) and corresponding hematoxylin and eosin (HE) staining (**e**–**h**), macrophage staining (**i**–**l**), and Masson-Trichrome staining (**m**–**p**) are shown. The yellow break lines in macro view (**a**–**d**) show the cutting lines. The high-magnification images are displayed as a picture-in-picture and macrophage stains as brown spots (white arrow). Scale bar = 100 µm for X20 figures, and 50 µm for X40 figures.

All mice survived after the injury. In the macro view, two (17%) of 12 sheets in the MHS group were partially detached from the liver surface due to invasion of the connective tissue, while 12 (100%) of 12 sheets in the microneedle-less mesh group had completely migrated into the greater omentum tissues. One migration sheet was found at 3 weeks and the other at 8 weeks. The migration rate was significantly lower in the MHS group (*p* < 0.01). Figure 4 shows the pathological changes in the MHS after implantation. Macroscopic examinations showed that the MHS started to be degraded at 3 weeks and was almost invisible at 30 weeks (Fig. 4a–d). H&E-stained slides (Fig. 4e–h) show inflammatory cell infiltration around the microneedles at 3 weeks (Fig. 4e), which remained around the basement site of the microneedles at 8 weeks (Fig. 4f). At 18 weeks, the microneedle disappeared and only the lattice of the MHS was observed (Fig. 4g). At 30 weeks, the lattice was surrounded by liver cells (Fig. 4h). Macrophage-stained slides (Fig. 4i–l) show that massive macrophage infiltration was observed around the microneedles at 8 weeks (Fig. 4j) and existed near the lattice even at 18 weeks (Fig. 4k). Macrophages are rarely seen at 30 weeks (Fig. 4k). Masson trichrome staining examinations (Fig. 4m–p) showed that the collagen fibers appeared around the microneedle from 3 weeks and (Fig. 4m) and still surrounded the lattice (Fig. 4p). No giant cells and granuloma were observed during the entire observation period.

### Quantitative pathological assessment

**Figure 5:**
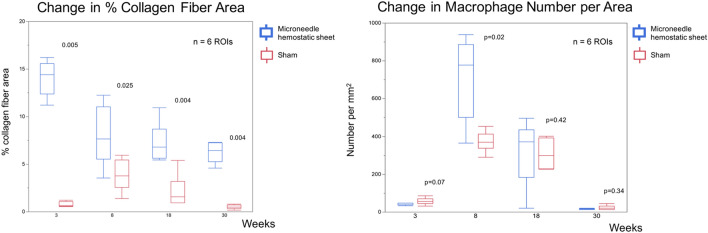
Quantitative pathological assessment.

Figure 5 shows the results of quantitative pathological assessment of the microneedle and sham groups at each time point. The number of macrophages per area in the microneedle group tended to be lower than that in the sham group at 3 weeks (4.7 [39.4–45.7] vs. 56.3 [45.7–70.0], *p* = 0.07) but was significantly higher at 8 weeks (778.2 [500.7–887.7] vs. 369.1 [337.0–413.3], *p* = 0.02) and was comparable at 18 and 30 weeks (microneedle 371.9 [184.7–436.4] vs. sham 299.0 [229.1–391.5], *p* = 0.42 and microneedle 16.7 [14.0–18.8] vs. sham 20.1 [14.4–31.1], *p* = 0.34).

The % collagen fiber area of the MHS group was significantly higher than that of the sham group at each time point (3 weeks, 14.4 [12.4–15.6]% vs. 0.69 [0.6–1.1]%, *p* = 0.005; 8 weeks, 7.6 [5.5–11.0]% vs. 3.8 [2.6–5.4]%, *p* = 0.025; 18 weeks, 6.9 [5.6–8.7]%, vs. 1.6 [0.96–3.2]%, *p* = 0.004; 30 weeks, 6.5 [5.3–7.3]% vs. 0.5 [0.3–0.7] %, *p* = 0.004).

Quantitative pathological assessments of the microneedle hemostatic sheet (MHS) and sham groups at each point. The number of macrophages per area of the MHS group tended to be lower than that of the sham group at 3 weeks (4.7 [39.4–45.7] vs. 56.3 [45.7–70.0], *p* = 0.07). At 8 weeks, the number of macrophages per area of the MHS group was significantly higher than that of the sham group (778.2 [500.7–887.7] vs. 369.1 [337.0–413.3], *p* = 0.02). At 18 weeks, the numbers of macrophages per area were similar between the two groups (MHS 371.9 [184.7–436.4] vs. sham 299.0 [229.1–391.5], *p* = 0.42). The numbers of macrophages per area were also similar at 30 weeks (MHS 16.7 [14.0–18.8] vs. sham 20.1 [14.4–31.1], *p* = 0.34). The % collagen fibers area of the MHS group was significantly higher than that of the sham group at each point (3 weeks, 14.4 [12.4–15.6] % vs. 0.69 [0.6–1.1] %, *p* = 0.005; 8 weeks 7.6 [5.5–11.0] %, vs. 3.8 [2.6–5.4] %, *p* = 0.025; 18 weeks, 6.9 [5.6–8.7] % vs. 1.6 [0.96–3.2] %, *p* = 0.004; 30 weeks, 6.5 [5.3–7.3] %, vs. 0.5 [0.3–0.7] %, *p* = 0.004).

### Optical coherence tomography imaging of the MHS on human aorta

**Figure 6:**
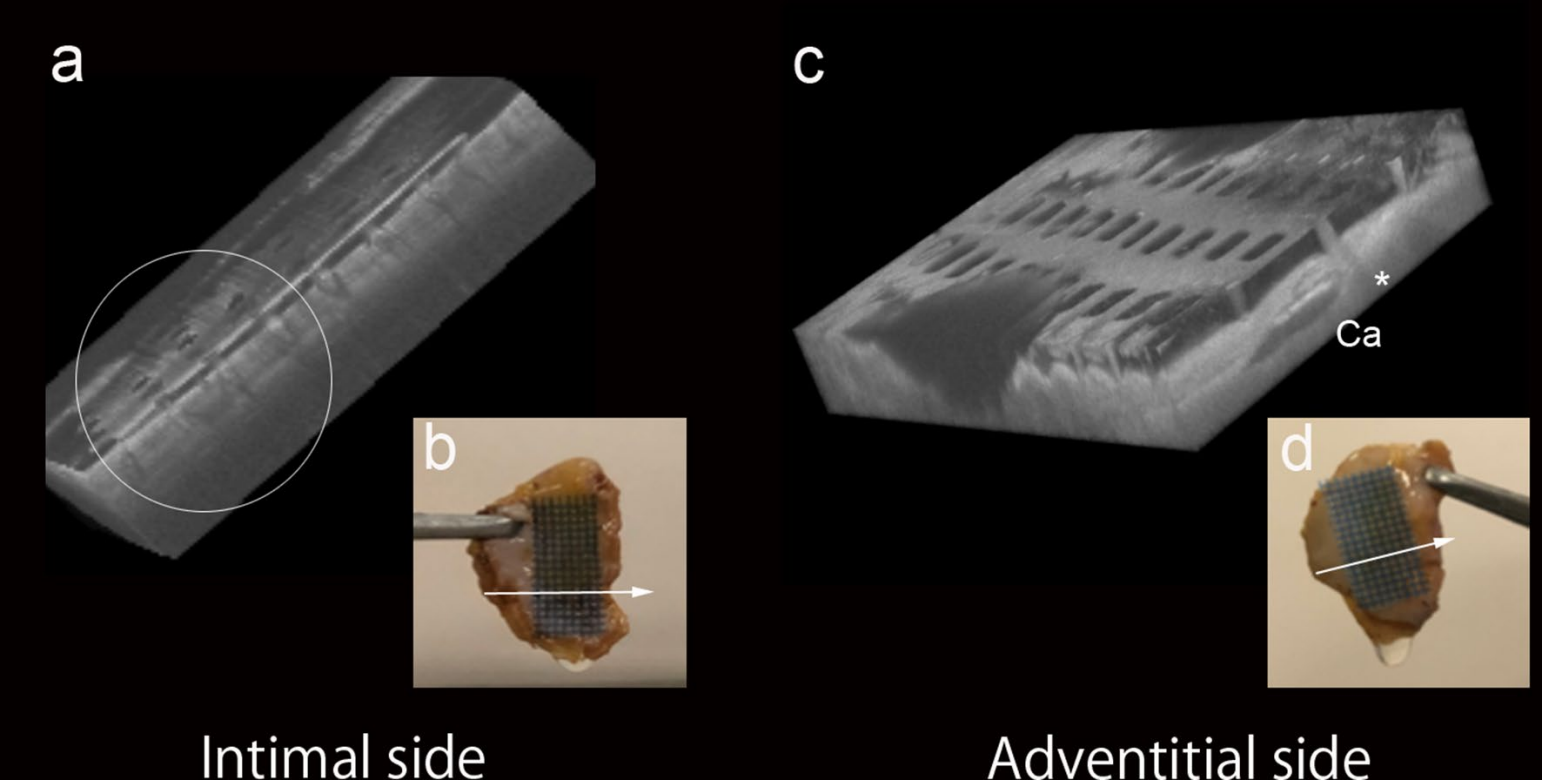
Optical coherence tomography imaging of the microneedle hemostatic sheet on human aorta. Volume rendering images and macro images of the microneedle hemostatic sheet (MHS) on the intimal side (**a** and **b**) and adventitial side (**c** and **d**) of the human aorta, respectively. (**a**) Optical coherence tomography (OCT) shows that the microneedles grab and spread out the tissue and close spaces between the tissue surface and the MHS. The MHS tracks the undulation of the human aorta surface (white circle). (**c**) The MHS pierces the adventitial wall (*), even if a calcified plaque (Ca) exists. The macro images show that the MHS fixes on the human aorta even under wet conditions. The white arrow indicates the imaging direction.

All MHSs were successfully attached to the human aorta. Figure 6 shows the optical coherence tomography images of the MHS on both sides of the human aorta. The MHS could track the undulation of the human aorta’s luminal surface while the microneedles grab and spread the tissue. Moreover, a small space between the tissue surface and microneedle sheet was observed (Fig. 6a). It was noted that the MHS pierces the adventitial wall and it still does so even if a calcified plaque is present (Fig. 6c). The MHS could be firmly fixed even under wet conditions (Fig. 6b,d).

### Swine model for left ventricular wall

**Figure 7:**
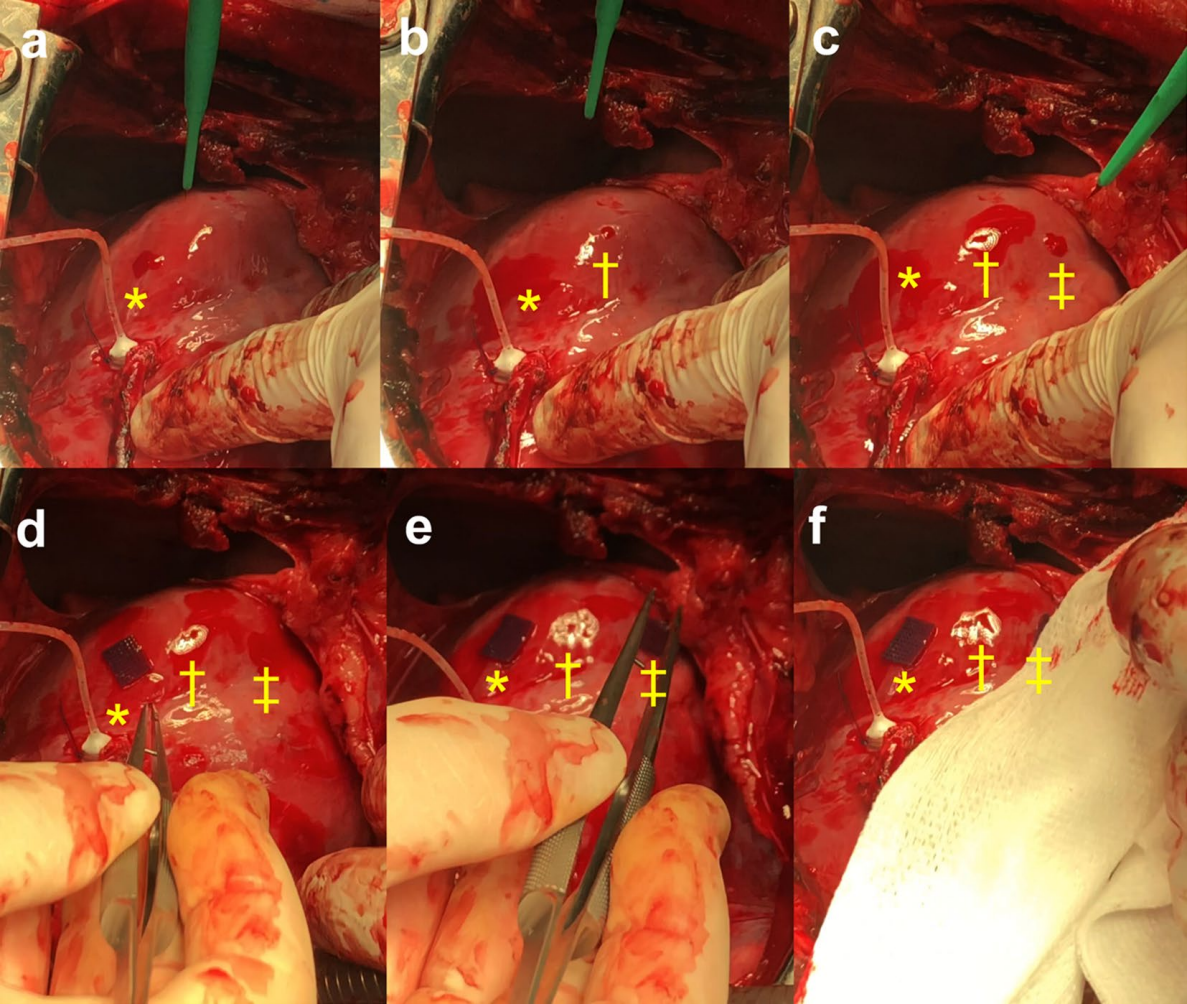
Microneedle hemostatic sheet in a living on-pump bypass surgery swine model. (**a**–**c**) First, we quickly made three cut wounds (*, †, ‡) on the left ventricular wall and confirmed bleeding from the wounds. (**d**)We next placed the microneedle sheet on to one wound site (*). The microneedle hemostatic sheets were easily attached to the left ventricular wall and fixed against a vigorous wall motion. A few seconds after attaching the microneedle hemostatic sheet onto the wound site, bleeding was stopped even under heparinization. (**e**) In contrast, bleeding continued from the control site (†) through the study. (**f**) The microneedle hemostatic also serves as a good un-slipping scaffold for the gauze compression hemostatic procedure (‡).

A pilot study was performed on a swine model to confirm whether the MHS could work in a large living animal model that mimicked the human body. After confirmation of bleeding from the stabbed sites (Fig. 7a–c), we attached the MHS to one stabbed site (Fig. 7d*). The MHS was easily attached to the left ventricular wall and remained fixed against vigorous wall motion. The bleeding was suspended within seconds even under heparinization (Fig. 7d). In contrast, bleeding continued from the control site (†) (Fig. 7e). We also confirmed that the MHS could serve as a good non-slipping scaffold for the gauze compression procedure that was the usual surgeon's technique for hemostasis (Fig. 7f, ‡).

## Discussion

### New concept of microneedle technology

In this study, we firstly developed and proofed a new concept of a biodegradable hemostatic sheet based on microneedle technology. The unique features of our MHS allow its use for intracorporeal topical hemostatic purposes without abnormal wound healing.

Microneedles were first described in a study conducted in 1998 on transdermal drug delivery in which microneedles were demonstrated to penetrate human skin^7^. Since then, microneedle technology has attracted extensive scientific and industrial interests due to its properties including painless penetration, relative safety, and low cost^8^. The postulated widespread applications of microneedle sheets include the delivery of insulin, vaccines, anti-inflammatory substances, and other pharmaceuticals^9^. While microneedle sheets are expected to become a highly efficient, patient-friendly, and versatile device^10^, most purported applications are extracorporeal or an intradermal. In this study, we first proposed the intracorporeal application of microneedle technology for topical hemostasis. While dissolvable microneedles have previously been proposed as an innovative vaccine delivery platform^11^, the biodegradable part is limited to the microneedles themselves. In contrast, all parts of our microneedle sheet are constructed of biodegradable materials, which may allow its use in the living body. The enhanced inflammatory process and increased collagen fiber production at 3 weeks after microneedle sheet implantation have been gradually degraded over 30 weeks. This is consistent with a previous study showing that a coronary stent made of PLA degraded around 8 months after implantation^12^. The macrophage density at 3 weeks in the MHS group tended to be lower than that in the sham group, while it reversed at 8 weeks, suggesting that two different healing processes had occurred in which macrophages were involved: one possibly for the healing process of the stabbed injury followed by PLA absorption. In the MHS group, mechanical hemostasis by the MHS could minimize the stabbed injury, resulting in low-grade inflammation as compared with that in the sham group in the early phase. Subsequently, the PLA absorption led to another inflammation process. Therefore, the macrophage density would be reversed. The degrees of macrophage infiltration were comparable between the MHS and sham groups at 18 weeks.

In addition, our MHS is flexible enough for use in the living body. The flexibility was comparable to that of bonding surgical tape. This flexibility seems to be based on its mesh structure. The MHS could be used with forceps in surgical procedures. Furthermore, we proved that the biodegradable microneedles had sufficient strength to penetrate the swine left ventricular wall and both sides of the human aorta. These findings warrant future studies to assess the use of the MHS in cardiovascular surgery.

The main function of our microneedle sheet was topical hemostasis. Compared to the microneedle-less mesh sheet and sham groups, the bleeding time in the cut wound model was reduced by use of the MHS alone and without the use of additional hemostatic drugs. No differences were observed in bleeding times between the microneedle-less mesh sheet and sham groups in the cut wound model. These findings indicate that the microneedles themselves play a role in the hemostatic process. The optical coherence tomography images showed that the microneedles pierced and spread the surrounding tissue. This effect may spread the tissue and compress the wound from all sides, working as a surgical stapler^13^ that is superior to vascular sealing devices in reducing blood loss^14^. Furthermore, very small spaces were observed between the tissue surface and the microneedle sheet. These spaces may act as a scaffold for native platelet aggregation and blood coagulation systems. Further studies are needed to clarify the detailed mechanisms by which hemostasis is achieved by use of the microneedle hemostatic sheet. Regardless of the mechanism, our technology contributes to hemostatic procedures and extends the application of microneedle technology.

### Potential advantages of the MHS

The MHS has some potential advantages compared to current hemostatic devices. First, there is no increase in infectious risk. Some topical hemostatic devices use human blood-derived materials such as human fibrinogen and thrombin, whereas the microneedle sheet is made of PLA. The low manufacturing cost is also an advantage. Furthermore, the microneedle sheets are ready to use without any special storage requirements. In this study, we used the MHS that had been stored in a cell culture dish at room temperature without a desiccant for 1–2 months. Easy handling is also noted. While human fibrinogen and thrombin are very powerful and natural hemostatic agents, they generally take around 10 min to provide hemostasis and adhesion^4^. During that time, surgeons need to fix the hemostatic products onto very slimy bleeding tissue with their own hands. In contrast, the MHS is instantly attached to tissue and also offers easy repositioning and removal. In cases with inadequate placement, surgeons can remove or reposition the MHS. Finally, the MHS can be used to assist in other hemostatic procedures such as gauze compression.

### Clinical application

One potential application of the MHS is topical hemostasis for parenchymatous organs, large vessels, and heart wall during trauma or surgery. The features of our technology, including being firmly adhesive and easily removable, biodegradable, and flexible, have the potential to make it easier for surgeons to stop bleeding from internal organs even when they are in a wet condition. Based on its good flexibility, the MHS could be rolled into small tubes and used in laparoscopic and endoscope surgeries. Needless to say, further investigation should be performed regarding the safety data for endovascular usage of the MHS, and the draining and absorption mechanism of PLA in the vascular wall if the MHS is applied to the vascular system. As drug delivery was the original role of microneedle technology, our sheet may also have potential for slow drug release in living bodies if drugs are combined with PLA.

Considering the commercialization of the MHS, the current production method would become a limitation. The current production method can manufacture the MHS at a speed of 50 sheets per hour. The process of microneedle imprinting on the sheet is especially cumbersome. The roll-to-roll manufacturing process could be applied to imprint the microneedles on the sheet because our production method is based on thermal imprinting. This would fit the mass production requirements of the MHS. Further studies are needed for the evaluation of the same. Our MHS production process uses 3D printing technology. Sterility is an important issue when we apply the MHS in the human body, because most 3D printed products cannot be autoclaved^15^. It has been reported that ethylene oxide, hydrogen peroxide plasma, electron beam radiation, and gamma radiation can be applied for the sterilization of PLA materials^16^. With respect to high temperature, which is a classic sterilization method, the extrusion temperature used in our 3D printing is 130 °C, which is significantly higher than the temperature used in most autoclave cycles (typically 121 °C). This led us to think that our 3D printing itself would be an intrinsically sterile process^15^. In any case, we should clarify the best sterilization method for the MHS. In this swine study, the MHS was could be attached to the swine heart without any difficulty. However, an applicator would be helpful to put the MHS with quantitative force. Increasing the size and determining the ideal height of the microneedles for each organ is also a technical challenge when we prepare to use the MHS in the human body.

## Methods

### Flexibility measurement

**Figure 8:**
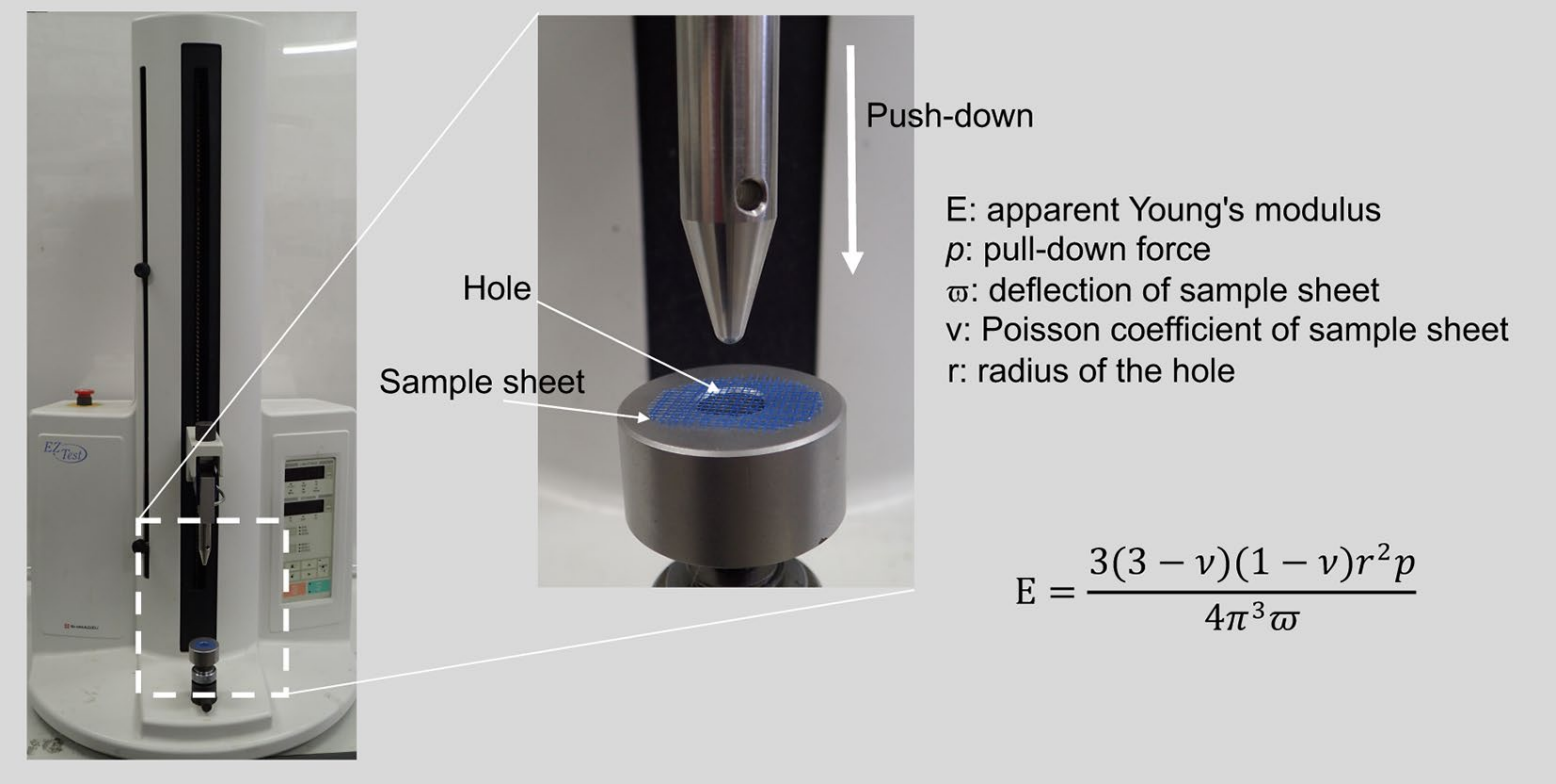
Method for flexibility measurement. The tensile tester has a table with a hole. The diameter of the hole (2r) is smaller than the diameter of the sheet. The center of a sample sheet is aligned with the center of the hole and placed onto the table. A rod is push down on the sample sheet. When the push-down force is *p,* the deflection of the sample sheet is ${\varpi }$, and the Poisson ratio of the sample sheet is ν, the apparent Young's modulus (E) is described as a shown.

A three-point bending test is commonly used to assess the elastic modulus of polymer materials. Beam shaped materials are suitable for the three-point bending test. However, the MHS was too thin to conduct the three-point bending test. Instead, we applied the apparent Young's modulus^17^ measured using a tensile tester (EZ-Test, Shimadzu Co, Ltd. Kyoto, Japan). The tensile tester has a table with a hole with a diameter (2r) smaller than the diameter of the sheet (Fig. 8). The center of a sample sheet was aligned with the center of the hole and placed on the table. A rod was push down on the sample sheet. For a push-down force *p,* sample sheet deflection ${\varpi }$, and sample sheet Poisson ratio of ν, the apparent Young's modulus (E) is described as follows.

$$ {\text{E}} = \frac{{3\left( {3 - \nu } \right)\left( {1 - \nu } \right)r^{2} p}}{{4\pi^{3} \varpi }} $$

We prepared two controls. One was a 200 µm-thick plane sheet of PLA with microneedles to investigate the effect of the mesh structure on the flexibility. The other was a bonding surgical tape to compare to a well-known material used in surgical procedures. To make the bonding surgical tape, we affixed adhesive sides of two sheets of surgical tape (Transpore Surgical Tape 1527-1, 3M Company, Saint Paul, MN, USA) together and cut out in 2 cm × 2 cm. We measured the apparent Young's modulus of the microneedle hemostatic sheet, the plane sheet with microneedles, and the bonding surgical tape five times each.

### Assessment of hemostatic time

All animal studies were approved by the Institutional Animal Care and Use Committees of the Wakayama Medical University. All methods were carried out in accordance with relevant guidelines and regulations. Pathogen-free 8-week-old male BALB/c mice were housed individually in cages under specific pathogen-free conditions during the experiments. The mice were anesthetized using a 4% isoflurane induction, followed by maintenance anesthesia with 1.5% isoflurane. After shaving, an incision was made along the abdominal midline to expose the liver.

A surgical knife (No 11, FEATHER Safety Razor Co., Ltd. Osaka, Japan) was used to make a 2-mm wide × 2-mm deep wound. The knife had a triangular blade with a sharp point and flat cutting edge parallel to the handle and flat back for precision cutting, stripping, and sharp-angle cuts. Immediately after confirming the bleeding, the MHS was placed on the wound (microneedle group, n = 12). To assess the effect of microneedles themselves on hemostasis, a mesh sheet without microneedles that was made using the same process as that for the MHS was placed onto the wound (microneedle-less group, n = 12). The control group received neither wound treatment (sham group, n = 12). We placed a filter paper (No. 50, ADVANTEC, Tokyo, Japan) 5–10 mm away from the incision site. When the absorption of blood by the filter paper was suspended, we objectively confirmed the cessation of blood discharge. The hemostatic time was defined as the time required for no blood discharge from the incision site.

### Histopathological assessment

The mice were maintained and euthanized in at 3, 8, 18, and 30 weeks after the hemostatic time study and their livers were resected. At each resection time point, we visually assessed the migration of the MHS. The pathological samples were fixed in 4% formaldehyde with phosphate-buffered saline (PBS) (pH 7.2) and then embedded with paraffin. The sample was sliced including the intersection point of the MHS that should embed the microneedles. Sections 6-μm in thickness were stained with hematoxylin and eosin (H&E) and Masson's trichrome for histopathological analysis. Other sections were further processed for immunohistochemical analyses to evaluate macrophage infiltration. Two independent pathologists (Y. I and T.K) without any prior knowledge of the experimental procedures evaluated the histopathological and immunohistochemical findings.

Immunohistochemical analysis using anti-F4/80 antibody (Cosmo Bio Co., Ltd, Tokyo, Japan) was conducted to identify macrophage localization. The macrophages were stained as brown spots. Every time point (3 weeks, 8 weeks, 18 weeks, and 30 weeks) and the 6 regions of interest (ROIs) were randomly set for the visible liver tissue at 200 × magnification, in which the liver surface and the microneedles were included^18^. The number of macrophages in the ROI were automatically counted in ImageJ. The number of macrophages per area was calculated using Eq. (1).

$$ Macrophage\;number\;per\;area = \frac{Macrophage\;number}{{ROI \;area}} $$

According to a previous study of wound healing^6^, Masson's trichrome stain histological data were digitalized and transferred to ImageJ (National Institutes of Health, Bethesda, MD, USA). As with macrophages per area analysis, the ROI was set for visible liver tissue at 20 × magnification. The collagen fibre areas were calculated using ImageJ. The % collagen fiber area was calculated using Eq. (2).

$$ \% collagen\;fiber\;area = \frac{Collagen\;fiber\;area}{{ROI\;area}}*100 $$

### Optical coherence tomography imaging study

This study was approved by the Ethics Committee of Wakayama Medical University (approval number: 2442), and followed all applicable institutional and national guidelines for the care and use of human materials. Informed consent was obtained from legally authorized representative. One of the potential applications of the MHS is for surgical use for vascular hemostasis, followed by endovascular usage. To clarify whether the MHS could be placed on the artery, we placed the MHS on both sides of the aorta. Within 24 h of death, a total of two aorta samples 10 mm in length were resected from the cadavers. Two aorta samples were cut into four half-pipes and stored in a PBS-filled tray. The slightly moistened half-pipe samples were removed from the tray and the MHS was placed on the luminal site. An intravascular frequency-domain optical coherence tomography catheter (LUNAWAVE, Terumo, Tokyo, Japan) was then placed on the MHS and imaging was performed in air with a 5 mm/sec motorized pull-back. The imaging datasets were transferred to ImageJ for analysis.

### Swine left ventricular wall model

A pilot study was performed on a swine model to confirm whether the MHS could work in a large living animal model that mimicked the human body. All animal studies were approved by the Institutional Animal Care and Use Committees of the Wakayama Medical University. All methods were carried out in accordance with relevant guidelines and regulations. A female swine weighing 50.5 kg and fed a cholesterol-free diet was used humanely. Anesthesia was performed with an intramuscular injection of ketamine (20 mg/kg), atropine (1 mg), and pentobarbital sodium (30 mg/kg) via the internal jugular vein. After endotracheal intubation, mechanical positive pressure ventilation was maintained with a mixture of oxygen and isoflurane at a tidal volume of 10 mL/kg. Isoflurane inhalation (0.5–5%) and a bolus injection of pentobarbital (25 mg/kg) were used for anesthesia. On-pump bypass surgery was performed using the standard procedure under heparinization (200 U/kg). Active clotting time was measured every 15 min and maintained for > 200 s. After completion of the on-pump bypass surgery, 3 incision sites were made on the left ventricular wall using a knife; one site for placing only the MHS, one for a control, and the other for placing the MHS with gauze compression, respectively. As per our surgeon’s opinion, the MHS size and figure should be fit on the incision size and figure. They cut the MHS into their desired size and figure from the original size of 2 cm × 2 cm square to approximately 8 mm × 10 mm rectangular. It was just fit to their finger pads. The MHS was placed onto the swine heart with a similar pushing force as the usual hemostasis procedure by gauze compression.

### Statistical analysis

Statistical analysis was performed using JMP version 14 for Mac (SAS Institute, Cary, NC, USA). Categorical variables are presented as numbers (%). Continuous variables are presented as means ± standard deviation. Shapiro–Wilk’s W tests were applied to evaluate normality. Student’s *t* tests were used for comparisons between the two groups and the Tukey–Kramer honestly significant difference method was used for multiple comparisons of normally distributed variables. Skewed variables were presented as medians [quartile]. Wilcoxon tests were applied for non-parametric comparisons between groups, while the Steel–Dwass test was applied for non-parametric multiple comparisons. For all analyses, *p* < 0.05 indicated statistical significance.

## Data Availability

All data generated or analysed during this study are included in this published article.
